# Suppression of Secondary Electron Emission from Nickel Surface by Graphene Composites Based on First-Principles Method

**DOI:** 10.3390/nano13182550

**Published:** 2023-09-12

**Authors:** Min Peng, Chang Nan, Dawei Wang, Meng Cao, Liang Zhang, Laijun Liu, Chunliang Liu, Dangqi Fang, Yiqi Zhang, Yonggui Zhai, Yongdong Li

**Affiliations:** 1 Key Laboratory for Physical Electronics and Devices of the Ministry of Education, School of Electronic Science and Engineering, Xi’an Jiaotong University, Xi’an 710049, China; L623121WXN@stu.xjtu.edu.cn (M.P.); mengcao@xjtu.exu.cn (M.C.); zhanglianghu@stu.xjtu.edu.cn (L.Z.); clliu@xjtu.edu.cn (C.L.); zhangyiqi@xjtu.edu.cn (Y.Z.); zhaiyg2021@xjtu.edu.cn (Y.Z.); 2 State Key Laboratory for Mechanical Behavior of Materials, School of Microelectronics, Xi’an Jiaotong University, Xi’an 710049, China; dawei.wang@xjtu.edu.cn; 3 Key Laboratory of Nonferrous Materials and New Processing Technology, College of Materials Science and Engineering, Guilin University of Technology, Guilin 541004, China; 2009011@glut.edu.cn; 4 MOE Key Laboratory for Nonequilibrium Synthesis and Modulation of Condensed Matter, School of Physics, Xi’an Jiaotong University, Xi’an 710049, China; fangdqphy@xjtu.edu.cn

**Keywords:** graphene, secondary electron yield (SEY), density functional theory (DFT), theoretical mechanism

## Abstract

Secondary electron emission (SEE) is a fundamental phenomenon of particle/surface interaction, and the multipactor effect induced by SEE can result in disastrous impacts on the performance of microwave devices. To suppress the SEE-induced multipactor, an Ni (111) surface covered with a monolayer of graphene was proposed and studied theoretically via the density functional theory (DFT) method. The calculation results indicated that redistribution of the electron density at the graphene/Ni (111) interface led to variations in the work function and the probability of SEE. To validate the theoretical results, experiments were performed to analyze secondary electron yield (SEY). The measurements showed a significant decrease in the SEY on an Ni (111) surface covered with a monolayer of graphene, accompanied by a decrease in the work function, which is consistent with the statistical evidence of a strong correlation between the work function and SEY of metals. A discussion was given on explaining the experimental phenomenon using theoretical calculation results, where the empty orbitals lead to an electron trapping effect, thereby reducing SEY.

## 1. Introduction

Secondary electron emission (SEE) under electron bombardment is a very important effect of electron solid interactions, and plays an important role in many fields of applied physics such as magnetron sputtering, plasma physics, spacecraft potential, gas discharge, scanning electron microscope, etc. [1,2,3,4,5,6]. For example, the multipactor [7,8] is a deleterious electron avalanche triggered by SEE, which is caused by the interaction of secondary electrons (SEs) with the alternating field of devices. The substantial bombardment of energetic particles on the walls of devices can degrade or even cause irreversible damage to the devices. This unwanted effect in some critical parts is difficult to avoid by proper RF field design. Therefore, reducing the secondary electron yield (SEY) of substrate materials is highly desired to suppress SEs multiplication in resonance with the RF field for aerospace applications.

To date, many investigations have been conducted to reduce the SEY to suppress multipactor effects. Mature techniques for low SEY mainly include surface roughening [9,10,11,12,13,14,15], coating with low-SEY films [16,17,18,19], and using low SEY materials as substitutes. For example, expensive inert metals (e.g., Au) and TiN coatings are commonly used to reduce SEY [17], thereby increasing the electric field threshold. Alodine, a conductive coating material that was first applied to the inner surface of space microwave devices to resist corrosion, has also been used to coat expensive inert metals to further reduce their SEY [18]. However, the above methods have their own limitations in application, and since the SEY of a material depends on many factors such as surface termination, surface morphology, and its electronic band structure, it is still highly desirable to develop guidelines to reduce the SEY of a material by simple surface modification. Carbon coatings, including amorphous carbon [20,21,22] and graphene [23,24,25,26,27,28], for SEY reduction have become a research hotspot. In particular, graphene has become popular for its remarkable electronic, mechanical, and thermal properties, and there were a lot of reports on the ultralow SEY of graphene. Montero et al. [29] used graphene nanoplatelets to study the SEE properties and focused on a novel strategy of graphene nanoplatelets with rough surface morphology for low SEY. This SEY reduction combined the known suppression effect from texture and roughness [30]. Bertoni et al. [31] presented a first-principles calculation of the atomic and electronic structure of the graphene/Ni (111) interface. Ligato et al. [32] studied the graphene on lattice-matched and lattice-mismatched Ni surfaces, and gave the two-dimensional character of the oscillating charge at the interface, which implies the possibility of SEE suppression via graphene/Ni (111) surface. However, the underlying mechanism of its SEE suppression is not clear yet. Moreover, experiments are lacking for validating the effectiveness of its SEE suppression.

In addition, as early as 1950, Baroody [33] pointed out that metals with larger work functions may be better emitters. For these metals, the essence that determines the SEY values as well as the correlation between the work function and its maximum SEY, is the behavior of electrons. In addition, surface modification can affect the work function [34,35] which, in turn, affects its SEY. Since the work function can be easily calculated for different surface conditions [36,37], a correlation between the work function and the maximum SEY will be useful in finding new materials and structures with low SEY.

Therefore, the aim of this work was to investigate the characters of the graphene/Ni (111) interface through theoretical calculations, and to design a feasible solution of low SEY based on this which could withstand the experimental verification. The greater expectation was to further obtain the underlying mechanisms and a practical law for seeking low SEY. Specifically, we were interested in how the calculated electronic properties affect the work function value and how this effect reflected on the SEY. Section 2 describes the methods that are used to achieve this work. We prepared a processing experiment to validate the results of the designed scheme of SEE suppression. Section 3 shows the SEE-involved electronic structural properties and the calculated work function on this interface, as well as the experimental observed decrease in SEY and work function. Section 4 discusses the correlation between work function and SEY of materials and elaborates on the underlying micro-mechanism, which offers an attempt to find out an exploratory approach to make surface modification using an easy-to-use indicator for SEY.

## 2. Materials and Methods

### 2.1. Theoretical Analysis and Design via DFT

**Figure 1:**
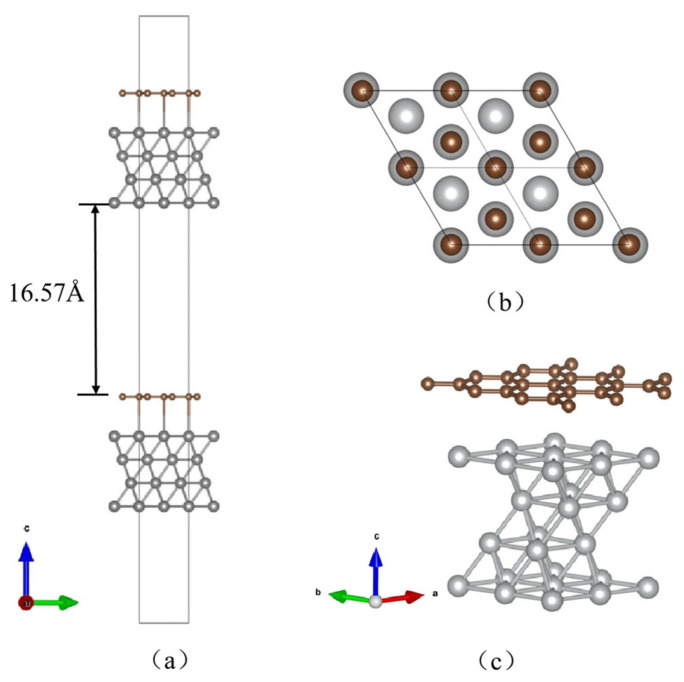
(**a**) The side view, (**b**) top view, and (**c**) three-dimensional supercell of the established slab for graphene/Ni (111) interface.

All DFT calculations in this work were performed using Vienna Ab-initio Simulation Package (VASP) within the generalized gradient approximation (GGA) [38,39] for the exchange and correlation energies. For design examples, the lattice constants of graphene and Ni (111) were 2.45 Å and 4.97 Å, respectively, and, based on this, the graphene/Ni (111) interface was modeled using a supercell constructed by standard unit cells of the two materials, as shown in Figure 1, with a mismatch degree of less than 1% for lattice adjustment.

The length of the vacuum gap was set to 16.57 Å, and the initial distance between surface nickel atoms and carbon atoms was 3 Å. With the convergence test, the first Brillouin zone was sampled using a *k*-point mesh of 15 × 15 × 1. The cut-off energy for the plane wave basis was 1.3 times the maximum energy value based on experiential rules and so it was set to 520 eV, and the energy convergence criterion was set to 10^−10^ Hartree. Ground state calculations and optimization of the studied systems were performed using the pseudopotentials of the Perdew–Burke–Ernzerhof functional with the projector augmented wave (PAW-PBE). In the structural relaxation, one layer of atoms at the bottom of the substrate metal was fixed to simulate the bulk material, whereas the three layers on the surface were released. Relaxation ended when the maximum value of the interatomic force was less than 0.02 eV/Å. Furthermore, the work function *φ* calculated in this study was obtained by self-consistent calculations, as given by:

(1) $$\varphi =E_{v}-E_{F}$$

where $E_{v}$ is the vacuum level energy obtained from the vacuum potential distribution, which was obtained by the *z*-axis averaging the electrostatic potential output LOCPOT, and $E_{F}$ is the Fermi energy obtained from calculation outputs in VASP. The established model could well describe the actual structural system and was used to conduct non-self-consistent calculations to obtain more information of electronic structure properties, e.g., the total electronic density of states (DOS) and partial density of states (PDOS) on the graphene/Ni (111) interface, providing a basis for designing suppression schemes of SEE.

### 2.2. Experiments for Material Preparing and Processing

**Figure 2:**
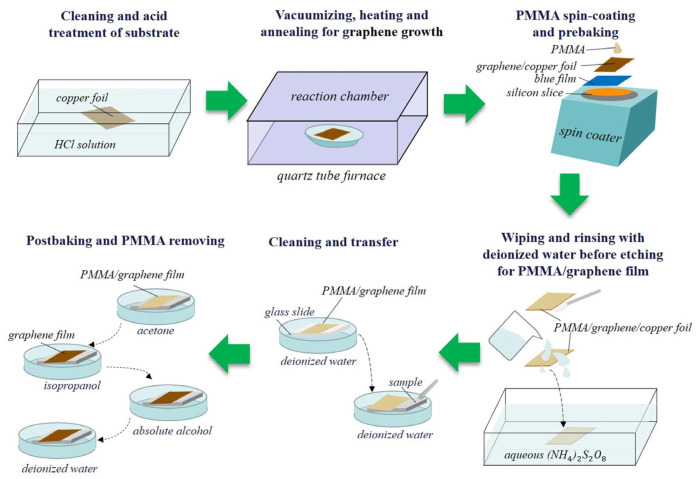
Schematic diagram of the preparation process of graphene covered samples.

Experiments were designed to verify the theoretical calculations. In the experimental preparation, single-crystal Ni (111) (cubic phase) samples were machined into dimensions of 10 mm × 10 mm × 1 mm in a class 1000 clean room (HEFEI KEJING, Hefei, China). The nominal uncertainty of these samples was ±3 Å in the crystal direction, and the surface roughness was less than 100 Å. Then, graphene was grown on copper foils (25-μm thick, 99.8%, Alfa Aesar, Haverhill, MA, USA) via low-pressure chemical vapor deposition and later transferred to the surface of the nickel samples. The specific steps of this process are shown in Figure 2, and additional operational details have been reported in a previous study [40].

The Ni (111) and graphene/Ni (111) surfaces were characterized by X-ray diffraction, a field emission scanning electron microscope (FE-SEM) (Gemini SEM500, ZEISS, Jena, Germany), and Raman spectrometry (HR800). The X-ray diffraction pattern was used to determine the crystalline phase of the nickel surface, and the SEM images were used to show the morphology of graphene on the Ni (111) surface. The Raman spectra of the graphene-covered samples were obtained using an excitation laser at 532 nm to investigate the monolayer graphene.

**Figure 3:**
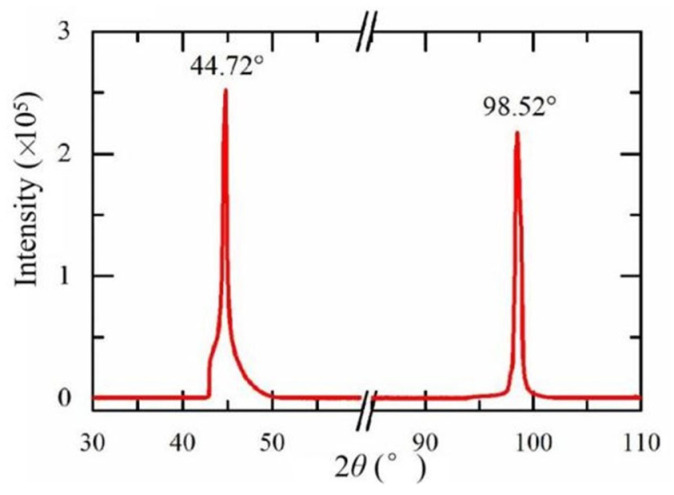
The X-ray diffraction pattern of the nickel sample surface.

**Figure 4:**
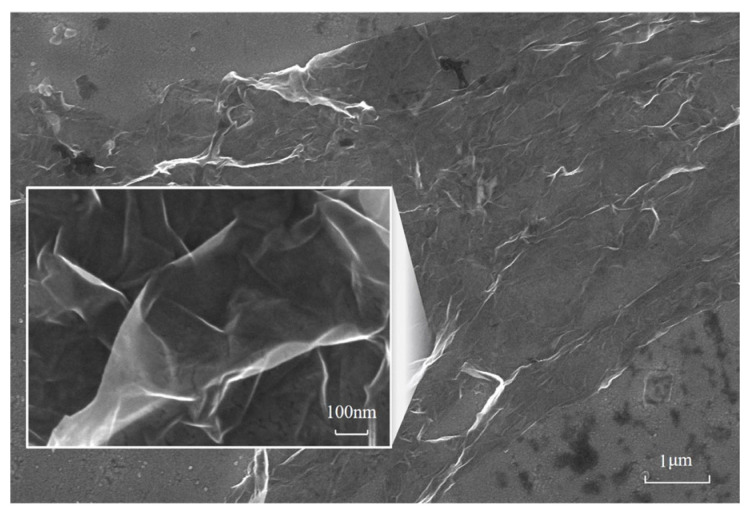
The SEM images of the graphene/Ni (111) sample.

**Figure 5:**
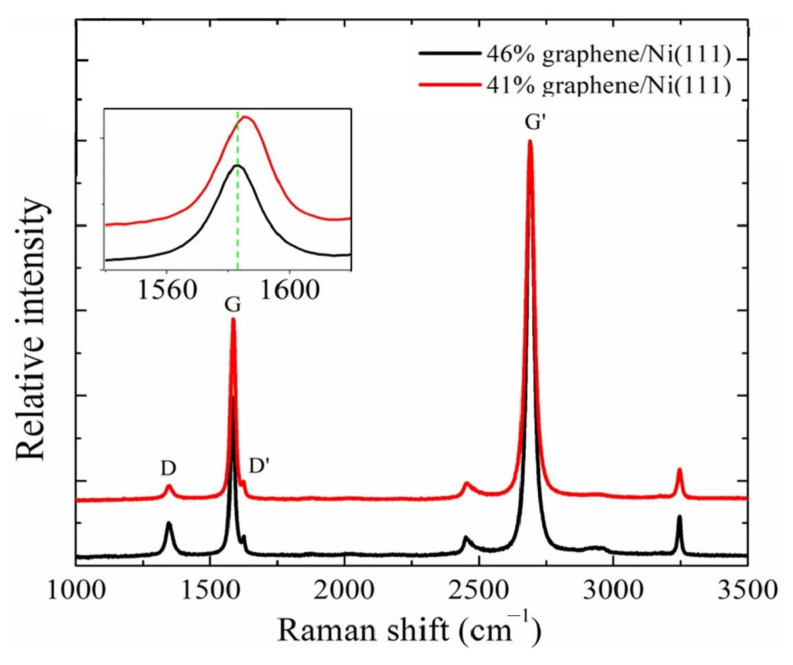
The Raman spectra from the graphene/Ni (111) samples.

The characterization results confirmed the applicability of the experimental samples. The crystal plane of the Ni (111) surface was confirmed by the characteristic angle of 44.7° in the X-ray diffraction pattern (Figure 3). Figure 4 shows the SEM image of the graphene/Ni (111) sample surface, where the proportion range of graphene coverage was 41–46%. The brighter area on the scale of 1μm was the nickel surface without graphene coverage, while the covered area appeared darker with wave-like bright textures caused by the transfer process, as shown in the magnified image on the scale of 100 nm. Figure 5 shows the Raman spectra of the samples used to verify the graphene. Since the metal has no Raman response due to a lack of dipole moment, these Raman signals must originate from the darker region on Ni (111). The two characteristic peaks of graphene are the G peak at 1583 cm^−1^ and the G’ peak at 2690 cm^−1^, which can be seen in the Figure, indicating the presence of graphene. The G peak can be slightly different for graphene on different substrates such as Si, glass, and NiFe [41,42,43], compared to free-standing graphene. The position of the G’ peak in Figure 5 essentially shows strain-free graphene [44], which is consistent with ab initio calculations within a small misfit strain of 0.67%. In addition, the profile of the G’ peak is a single Lorenz peak, indicating that the graphene is monolayer [45].

## 3. Theoretical and Experimental Results and Discussion

### 3.1. Explanation of SEE Suppression via DFT Simulation

**Figure 6:**
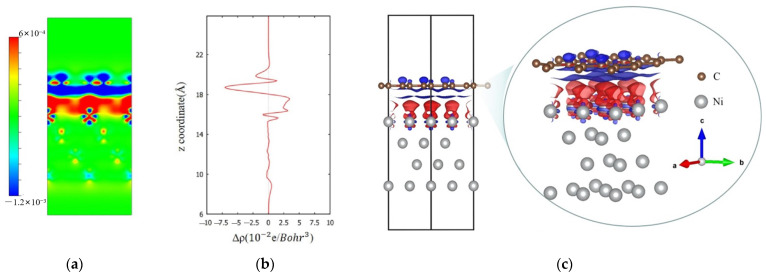
Diagrams of (**a**) two-dimensional section of differential charge density, (**b**) planar-averaged electron density difference ∆ρ(z) along the z direction, and (**c**) three-dimensional differential charge density for the graphene/Ni (111) interface.

Figure 6 shows the 2D and 3D distributions of charge densities of a supercell at the graphene/Ni (111) interface as well as the planar-averaged electron density difference ∆ρ(z) along the z direction, presented in Figure 6a, c, and b, respectively. The dark blue area in Figure 6a,c represents the space where the electron density decreases at the composite interface compared to the single bulk before, while the red area represents an increase. This means that the electron density of the outer orbitals of the nickel atoms near the interface increases, while the electron density of the carbon atoms near the vacuum layer decreases. This suggests that the outer shell electrons around the carbon atoms are transferred to the adjacent outer orbitals of the nickel atoms, resulting in more empty orbitals around the carbon atoms at the interface. These empty orbitals make a trapping effect to retain the outgoing SEs in the surface. In addition, the charge redistribution mentioned above generates a sort of equivalent dipole layer within a local space, which weakens the internal SEE below this local space, ultimately leading to a decrease in the probability of SEE. Therefore, this calculation result reveals that the scheme of composite structure can suppress SEE. As for the outgoing SEs whose energy are enough to overcome the blocking effect and enter the spatial range of this equivalent dipole layer in the shallow surface, they are accelerated by the built-in electric field from the equivalent dipole itself, which naturally corresponds to a reduction in the work function.

**Figure 7:**
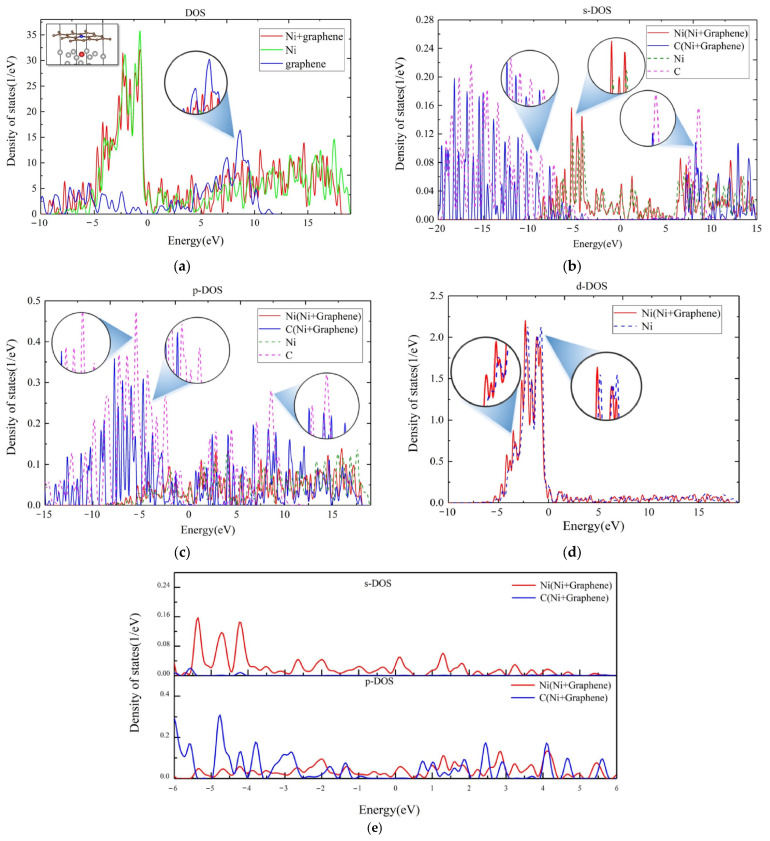
(**a**) The diagram of total DOS of calculated system; (**b**) the *s*-PDOS, (**c**) *p*-PDOS, and (**d**) *d*-PDOS diagrams of a single nickel atom in red and carbon atom in blue on the graphene/Ni (111) interface. (**e**) The detailed PDOS of the graphene/Ni (111) interface in the energy range of ±6 eV.

More results of DFT calculations will support this deduction as presented in Figure 7 and provide more microscopic evidence. Figure 7a shows the total DOS of all atoms in the calculated supercell. It was found that the peak in the 5–10 eV energy range disappeared after the composite, which is in consistent with the electron transfer track. For further details, Figure 7b, c and d present the PDOS for the s, p and d orbitals of the nickel atom, marked in red, and the carbon atom, marked in blue, in the Figure 7a, respectively. As shown in Figure 7b, the s-PDOS peaks of the carbon atom in the composite are lower than those of graphene for the energy beyond the range of about ±9 eV, while the density peaks of nickel atom in the composite are higher than those of simple metal Ni surface for the energy in the range of −9–13 eV. For the *p*-PDOS, the density peaks of the carbon atom in the composite were lower in the energy range of −15–10 eV, while the density peaks of the red nickel atom in the composite were higher in the energy range of 0–17 eV. For the *d*-PDOS from Figure 7d, there is little hybridization of the d orbitals, except for slight variations in the energy distribution of d orbitals for the nickel atom which are negligible. This shows that the *s*- and *p*-electrons are the main contributors to the charge transfer. To show more detail, Figure 7e presents the PDOS of graphene/Ni (111) interface in the energy range of ±6 eV. The energy band near the bottom of the conduction band is mainly composed of the *s* and *p* orbitals of Ni, as well as the *p* orbitals of carbon. This implies the hybridization between the nickel atom and the carbon atom, which contributes to the transfer of electrons.

**Figure 8:**
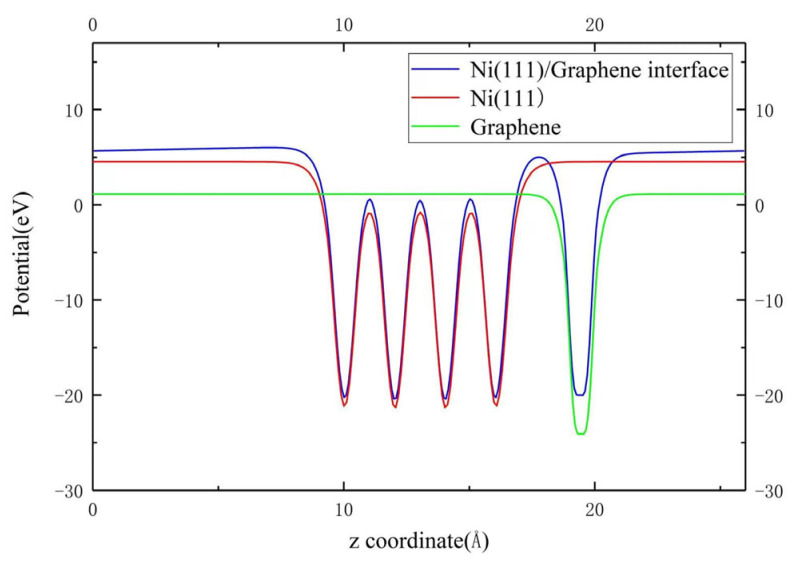
The comparison of vacuum barrier variation at the graphene/Ni (111) interface.

In addition, the redistribution of the electron density at the graphene/Ni (111) interface leads to a variation in the electrostatic potential, thus affecting the probability of electrons emitting and exiting the interface as well as the work function as Equation (1). Figure 8 shows the potential distributions of the vacuum layer before and after adsorption of graphene onto the Ni (111) surface, where the vacuum energy level of the composite structure can be given. This variation of potential barrier along the *z*-axis direction of the graphene/Ni (111) interface can be further used in a future correction of SEY Monte Carlo simulation. Considering the variation of fermi energy level after the composite, the work function *φ* can be calculated for this composite structure, as shown in the second column of Table 1.

### 3.2. Valilation of Suppressing SEE Methods

**Figure 9:**
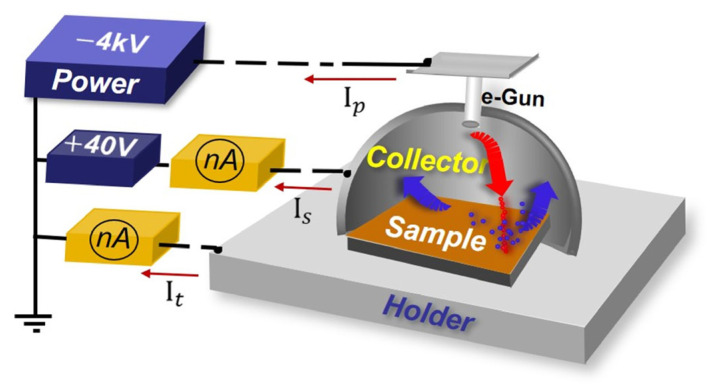
The diagrammatic sketch of the system for SEY measurement.

Work function and SEY were measured before and after the coverage. The SEY measurement was carried out using a home-made SEY system as outlined in Figure 9. The SEY values can be obtained using Equation (2), where the target current $I_{t}$ and secondary electrons current $I_{s}$ can be measured using the continuous irradiation, and the primary electron current ${I_{p}=I}_{t}+I_{s}$. For metal samples, the currents mentioned above are the measured conducting currents; for dielectric materials, the negative charge should accumulate on one side of the sample, while a corresponding positive charge density should form on the other side due to poor conductivity. The currents mentioned above refer to displacement currents, in this case, measured with a pulse method; this system can also be adapted for SEY measurement of insulators [46].

(2) $$\delta =\frac{I_{s}}{I_{p}}=\frac{I_{s}}{I_{t}+I_{s}}$$

The ultraviolet photoelectron spectra of the Ni (111) surface and the graphene covered ones were obtained using an ultraviolet photoelectron spectrometer (UPS) [47] (Thermo Fisher ESCALAB Xi+, Waltham, MA, USA) to determine the work function φ of the samples. These UPS measurements were performed with ultra-high vacuum (UHV) under the pressure of 10^−10^ mbar and the irradiation of 21.22 eV photons (He I line).

**Figure 10:**
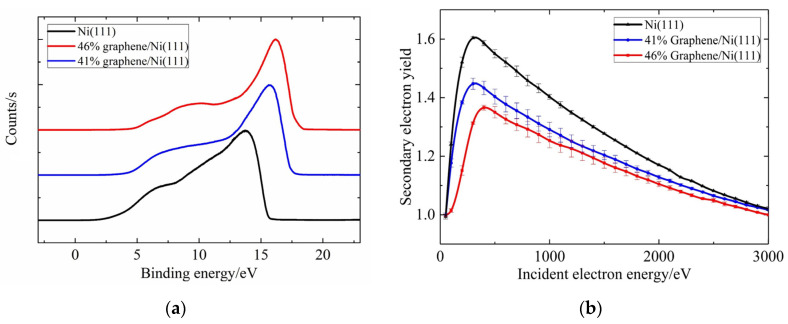
(**a**) The ultraviolet photoelectron spectra; (**b**) SEY curves for samples before and after graphene covered.

Figure 10 shows the measured spectra from the UPS and the SEY curves in the energy range of 50–3 keV for the three samples. Figure 10a shows that the 41 and 46% graphene/Ni (111) samples have a larger cut-off edge than the Ni (111) surface. Table 1 presents the experimental values of the work function obtained according to the UPS spectra in the third column, where the work function is reduced from 5.37 eV for the nickel surface to an average value around 3.42 eV for the graphene/Ni (111) samples, which had almost the same change trend with the calculated values within the error of 30%. Figure 10b shows the average SEY curve as a function of the primary electron energy for the samples, where a decrease in the maximum value of SEY is observed with the graphene covered on the surface. It was found that a larger proportion of graphene coverage led to a lower maximum SEY value, and the maximum SEY value after coverage is approximately 1.35. This declares that the experimental results well validate the effectiveness on suppressing the SEE of the composite structure designed according to theoretical calculations.

## 4. Discussion

**Figure 11:**
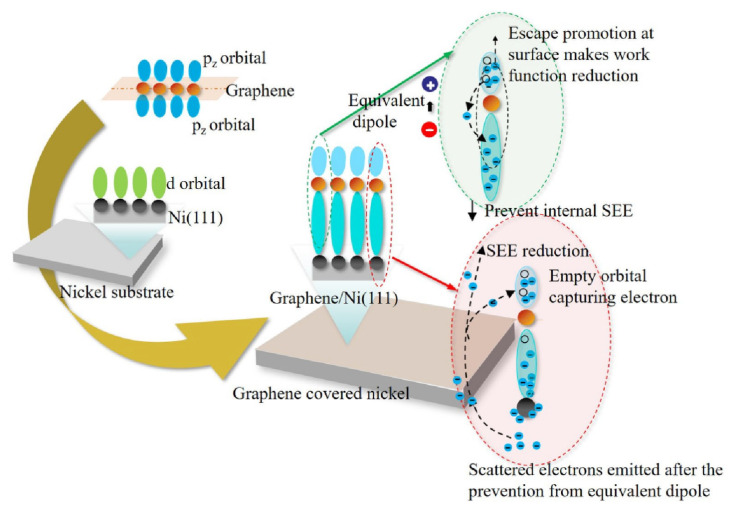
The schematic diagram of a mechanism of synchronous decrease of SEY and work function on the graphene covered nickel surface.

The synchronous reduction of SEY and work function on the graphene/Ni surface may contribute to an equivalent dipole layer formed by the charge transfer between graphene and nickel according to the calculation results. The hybridization of *p_x_*, and *p_y_* orbitals of nickel with *p_z_* orbitals of carbon induced a weak interaction between the atomic layers of carbon and nickel. A group of electrons migrated down from the carbon atoms to the nickel atoms, resulting in a downward shift of negative charge and leaving positive vacancies at the original positions. The charge transfer between the nickel and carbon atoms creates an equivalent dipole layer on the surface, as shown in the green dashed circle in Figure 11. This equivalent dipole layer resulted in a local electric field directed toward the inside of the material, which promoted the escape of electrons at the shallow surface, and thereby reduced the work function. Meanwhile, the equivalent dipole layer prevented the emission of inner SEs below the negative charge layer inside the material, where most SEs are generated with the range of tens of nanometers. Even if some secondary electrons excited by inelastic scattering have enough energy to overcome this negative charge layer, some of them would still be trapped by the vacancies and even empty orbitals of the monolayer carbon atoms. These trapped electrons would then be transported due to the π orbital as an intralaminar conduction of graphene rather than emitted. This explains why the SEY decreased, as shown in the red dashed circle in Figure 11. Thus, these SEs were more affected by the negative charge layer and this trapping effect of empty orbitals, which reduces the SEE. This is in accordance with the fact that the work function decreased while the SEE also decreased in the experiment. This indicates that this competitive factor outperformed the other one.

**Figure A1:**
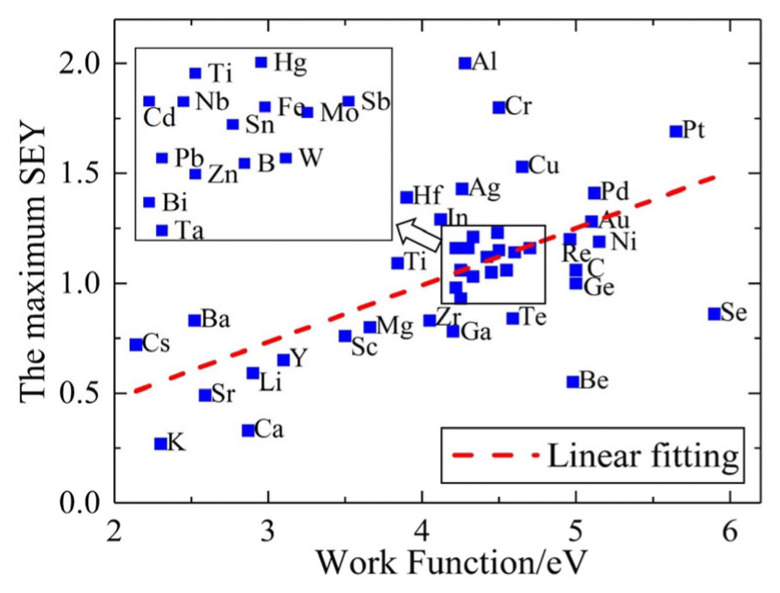
The correlation between the maximum SEY $\delta_{max}$ and work function *φ* values of different metals, which satisfies a fitting empirical equation: $\delta_{max}\approx 0.25\varphi$.

In this micro-mechanism, the work function *φ* can be used as a simplified indicator for the SEY on the surface owing to a concomitance of the reduction both of the work function and SEY. This is consistent with the previous findings in Ref [48] of a strong correlation between the work function and the maximum SEY of metals. We enhanced this correlation in Figure A1 by extending the data of 40 different metals which were obtained by Lin [49] et al. Thus, it is of great significance that the first-principles calculations based on periodic DFT can be applied to investigate the schemes of suppressing SEE from an atomistic perspective to design the structures or modifications on the surface.

## 5. Conclusions

In conclusion, the DFT calculations were conducted to design a graphene/Ni (111) composite interface to suppress SEY, which were validated by the results from an experimental study of surface modification with graphene on an Ni (111) surface. Moreover, the results of theoretical calculations provided a way to reveal the mechanism of SEE suppression and work function decrease on graphene/Ni (111) surfaces. A general strategy for achieving low SEY surface modification was summarized, which manifested that the work function *φ* can be used as a simplified indicator when evaluating surface SEY. Although we provided an insight into the mechanism, more abundant practical cases of surface modification are required to refine and strengthen the theory. In addition, the results of DFT calculations can also provide a basis for the first principles correction of MC simulations of SEY on the surface after surface modification [50,51,52]. This proposed mechanism supported by DFT calculations is of practical significance for a simplified expression of the correction methods in future MC simulations.

## Figures and Tables

**Table 1:** The experimental and calculated values of work function.

Material	Calculated Values of *φ* (eV)	Experimental Values of *φ* (eV)
Graphene	4.44	4.60
Ni (111)	5.11	5.37
Graphene/Ni (111)	3.73	3.42

## Data Availability

The data used to generate the curves shown are available on request.
